# Supplementary material for: Stiffness-Controlled Hydrogels for 3D Cell Culture Models
Source: Polymers (Basel). 2022 Dec 17;14(24):5530. doi: 10.3390/polym14245530 (PMC9786583; doi:10.3390/polym14245530)
Supplement: Supplementary file 1 [file polymers-14-05530-s001.zip › polymers-1990413-supplementary.pdf]

Supplementary material

Stiffness-Controlled Hydrogels for 3D Cell Culture Models

Arto Merivaara [1,*], Elle Koivunotko [1,†], Kalle Manninen [1,†], Tuomas Kaseva [2], Julia Monola [1], Eero Salli [2], Raili Koivuniemi [1], Sauli Savolainen [2,3], Sami Valkonen [1,4] and Marjo Yliperttula [1,*]

1 Drug Research Program, Division of Pharmaceutical Biosciences, Faculty of Pharmacy, University of Helsinki, 00014 Helsinki, Finland
2 HUS Medical Imaging Center, Radiology, University of Helsinki and Helsinki University Hospital, 00290 Helsinki, Finland
3 Department of Physics, University of Helsinki, 00014 Helsinki, Finland
4 School of Pharmacy, University of Eastern Finland, 70210 Kuopio, Finland
* Correspondence: arto.merivaara@helsinki.fi (A.M.); marjo.yliperttula@helsinki.fi (M.Y.); Tel.:+358-294-159-577 (A.M.); +358-294-159-141 (M.Y.)
† These authors contributed equally to this work.

1. SUPPLEMENTARY METHODS

1.1. Freeze-drying of NFC hydrogel

The freeze-drying cycle used in this study is shown in Figure S1. The samples were frozen at -47 °C, the primary drying was performed at -42 °C, and during the secondary drying, the temperature was ramped up 1 °C/min to 20 °C. The cycle length was 96 hours.

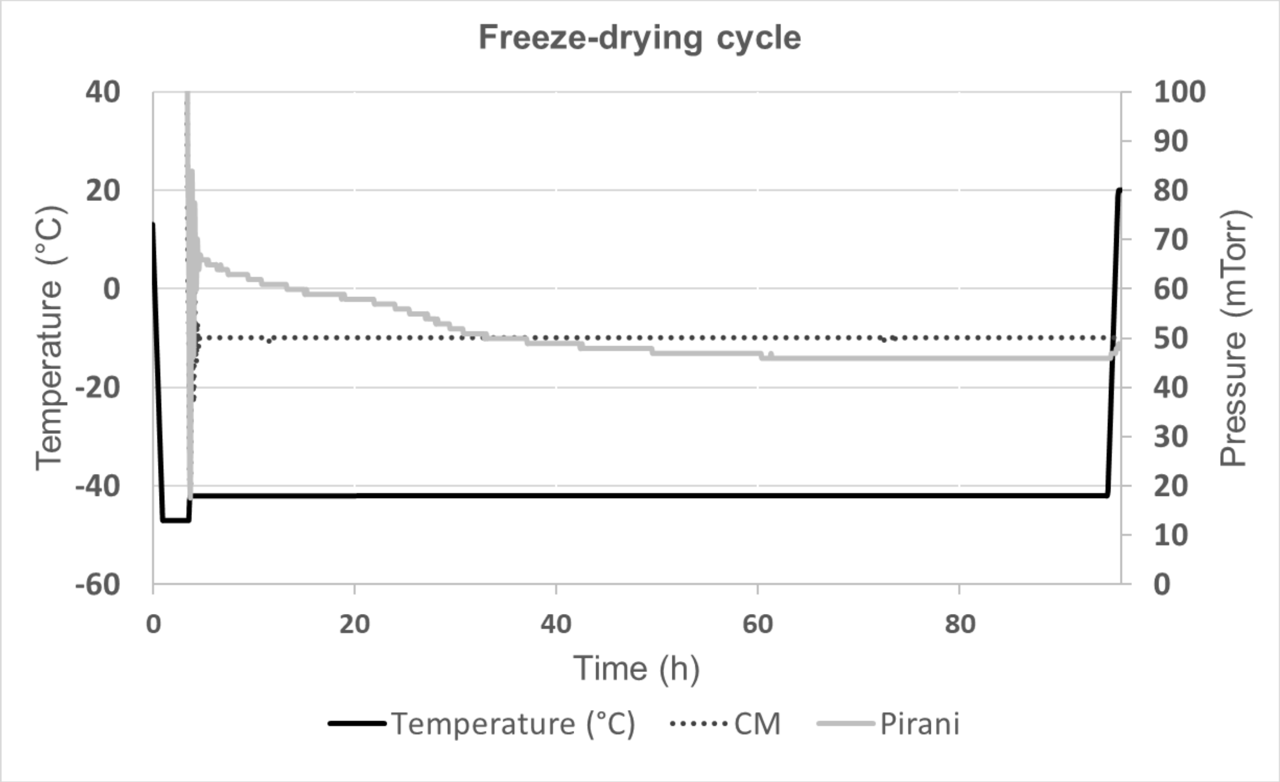

Figure S1. Freeze-drying cycle used in this study. The samples were frozen at -47 °C, the primary drying was performed at -42 °C and during the secondary drying, the temperature was ramped 1 °C/min to 20 °C. CM: Capacitance manometer.

2. SUPPLEMENTARY RESULTS

2.1. Freeze-drying and reconstitution of NFC hydrogel

The freeze-dried nanofibrillated cellulose (NFC) formed an elegant solid cake (Figure S2). After reconstitution with the water, a viscous NFC hydrogel was successfully formed.

For the samples reconstituted to different NFC concentrations than the original 1.5%, the reconstitution was performed with a mixture of water and specific cell medium to a wanted dilution.

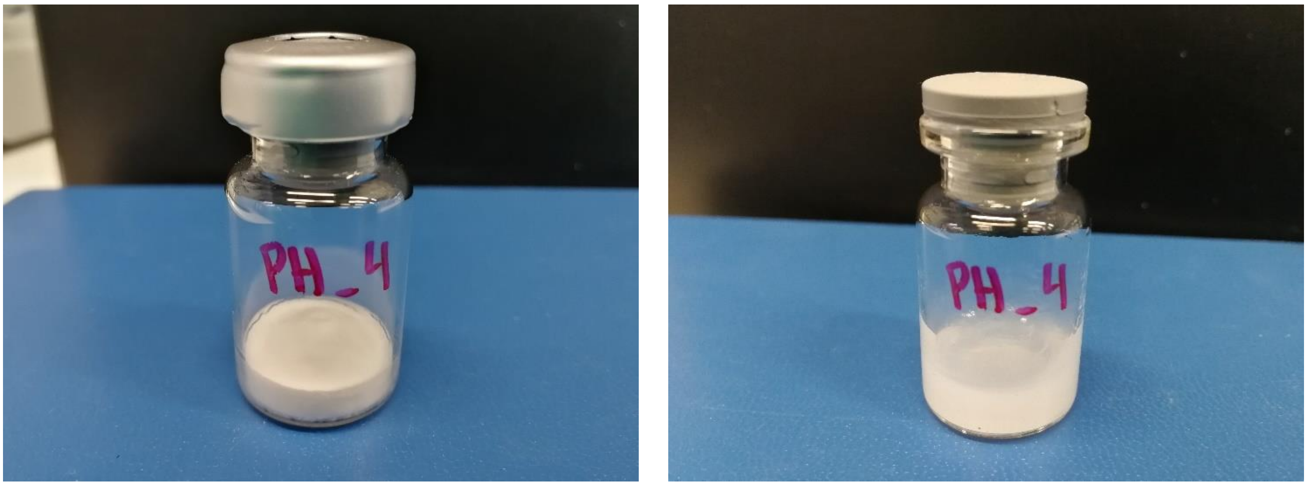

Figure S2. Freeze-dried (left) 1.5% nanofibrillated cellulose with 300 mM sucrose and reconstituted (right) 1.5% NFC hydrogel with 300 mM sucrose.

2.2. Rheological measurements of NFC hydrogel

The storage (G’) and loss moduli (G’’) of 3.0%, 1.5%, 1.0%, 0.8%, and 0.125% NFC hydrogels are reported in Figure S3.

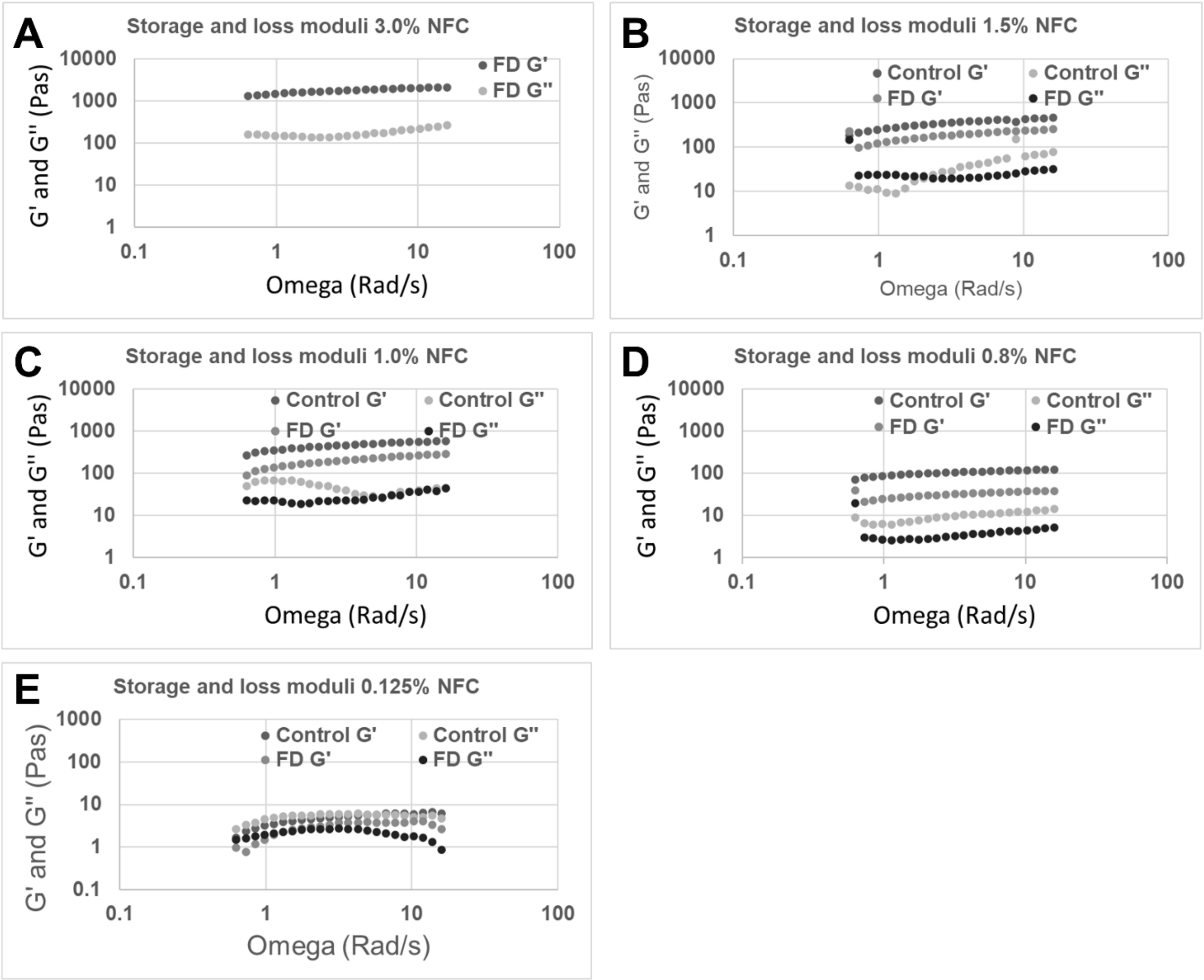

Figure S3. Storage (G`) and loss (G``) moduli of **(A)** 3.0%, **(B)** 1.5%, **(C)** 1.0%, **(D)** 0.8%, and **(E)** 0.125% NFC hydrogel.
